# Meta-analysis of the association between emphysematous change on thoracic computerized tomography scan and recurrent pneumothorax

**DOI:** 10.1093/qjmed/hcab020

**Published:** 2021-02-04

**Authors:** M Girish, P D Pharoah, S J Marciniak

**Affiliations:** From the Department of Respiratory Medicine, University of Cambridge, Addenbrooke's Hospital, Hills Rd, Cambridge CB2 0SP, UK; Department of Respiratory Medicine, Addenbrooke's Hospital, Cambridge University Hospitals NHS Foundation Trust, Cambridge Biomedical Campus, Hills Road, Cambridge CB2 0QQ, UK; Department of Oncology, University of Cambridge, Strangeways Research Laboratory, Worts Causeway, Cambridge CB1 8RN, UK; From the Department of Respiratory Medicine, University of Cambridge, Addenbrooke's Hospital, Hills Rd, Cambridge CB2 0SP, UK; Department of Respiratory Medicine, Addenbrooke's Hospital, Cambridge University Hospitals NHS Foundation Trust, Cambridge Biomedical Campus, Hills Road, Cambridge CB2 0QQ, UK; Cambridge Institute for Medical Research (CIMR), University of Cambridge, Hills Road, Cambridge CB2 0XY, UK

## Abstract

**Objectives:**

At least a third of patients go on to suffer a recurrence following a first spontaneous pneumothorax. Surgical intervention reduces the risk of recurrence and has been advocated as a primary treatment for pneumothorax. But surgery exposes patients to the risks of anaesthesia and in some cases can cause chronic pain. Risk stratification of patients to identify those most at risk of recurrence would help direct the most appropriate patients to early intervention. Many studies have addressed the role of thoracic computerized tomography (CT) in identifying those individuals at increased risk of recurrence, but a consensus is lacking.

**Aim:**

Our objective was to clarify whether CT provides valuable prognostic information for recurrent pneumothorax.

**Design:**

Meta-analysis.

**Methods:**

We conducted an exhaustive search of the literature for thoracic CT imaging and pneumothorax, and then performed a meta-analysis using a random effects model to estimate the common odds ratio and standard error.

**Results:**

Here, we show by meta-analysis of data from 2475 individuals that emphysematous change on CT scan is associated with a significant increased odds ratio for recurrent pneumothorax ipsilateral to the radiological abnormality (odds ratio 2.49, 95% confidence interval 1.51–4.13).

**Conclusions:**

The association holds true for primary spontaneous pneumothorax when considering emphysematous changes including blebs and bullae. Features, such as bullae at the azygoesophageal recess or increased Goddard score similarly predicted recurrent secondary pneumothorax, as shown by subgroup analysis. Our meta-analysis suggests that CT scanning has value in risk stratifying patients considering surgery for pneumothorax.

## Introduction

Spontaneous pneumothorax is a frequent presentation to respiratory services. In some cases, little or no intervention is required, but individuals with recurrent pneumothorax benefit from surgical intervention. Predicting who will suffer recurrences and therefore require pre-emptive surgery remains challenging.

The majority of spontaneous pneumothoraces appear to occur when cystic air spaces beneath the visceral pleura rupture.^1^ When smaller than 1–2 cm in diameter these lesions are called blebs, while larger subpleural cysts are called bullae. Emphysematous lesions, can occur in the otherwise healthy lungs of tall thin individuals, the asthenic habitus, and can rupture to cause primary spontaneous pneumothoraces (PSPs).^2^ By thoracic computerized tomography (CT), emphysematous lesions are seen in 80% of patients with PSP compared with 30% on healthy controls.^3^ When blebs and bullae form owing to an underlying pulmonary pathology, most frequently smoking-induced pulmonary emphysema, they can give rise to secondary spontaneous pneumothoraces (SSPs).^4^

The recurrence rate at 5 years following pneumothorax has been estimated to be around 30% overall, but 39% for those with underlying chronic lung disease.^5–7^ Although there are only limited data concerning risk of recurrence following a second pneumothorax,^5^ treatment guidelines recommend procedures to reduce the likelihood of further recurrences be considered.^8^^,^^9^ In paediatric practice, this tends to involve surgical removal of blebs and bullae, while in adults, this is combined with surgical obliteration of the pleural space by pleurectomy or talc poudrage.^8^ In those considered unfit for surgery, chemical pleurodesis with sclerosants is an option, most commonly using graded talc, but autologous blood is sometimes used. Some authors have advocated the use of pleurodesis as a primary intervention even following a first pneumothorax,^10^ but owing to the generally benign course of PSPs and the potential for operative complications including chronic pain, others recommend deferring surgery until a recurrence has occurred or if the initial air leak fails to resolve within a week.^8^^,^^9^

If recurrence could be accurately predicted, then primary surgery would be a more attractive option for selected patients. Underweight individuals appear to be at an increased risk of recurrence, but this is not highly discriminating in a population with typically low body mass indices.^11^ Whether radiological appearance can predict recurrence has remained controversial with reports suggesting a variety of potential prognostic features, while other reports suggest none exist.^12–15^ Cross-sectional imaging by CT can identify emphysematous lesions including blebs and bullae even when the plain chest X-ray appears normal.^3^^,^^16^ It is also noteworthy, that blebs and bullae seem not to account for all PSPs, with increased ‘pleural porosity’ having been proposed as an alternative cause.^1^^,^^17^

Current British Thoracic Society (BTS) guidance is that surgical intervention be offered following the first recurrence of a pneumothorax.^8^ Surgery following a first pneumothorax tends to be restricted to those who have suffered a tension pneumothorax or a persistent air leak, or individuals in high-risk occupations, such as pilots and divers. However, at present, prognostic features, e.g. CT appearances, are not part of these guidelines.

We performed a systematic review and meta-analysis of the published literature in order to clarify the association between the presence of blebs or bullae at the time of diagnosis of pneumothorax and recurrent pneumothorax. Our aim was to test the hypothesis that the presence of emphysematous changes on thoracic CT imaging was associated with increased likelihood of recurrent pneumothorax. We also wished to determine if laterality of recurrence, ipsilateral or contralateral, was associated with the laterality of the CT abnormalities. 

## Materials and methods

### Search strategy and selection criteria

We aimed to identify all peer-reviewed studies reporting on risk factors for recurrent pneumothorax in individuals who have had an initial pneumothorax. Preprints and conference proceedings were not included. EMBASE, MEDLINE and the Cochrane Library databases were interrogated up to 5 May 2020 using a formal search strategy. Initial Medical Subject Heading (MeSH) and other relevant terms were kept broad to retain any potentially relevant studies [CT OR ‘computerised tomography’ OR ‘computer assisted tomography’ OR ‘computed tomography’ OR ‘x ray computer assisted tomography’ OR ‘x ray computer assisted tomography’ (MeSH) AND pneumothorax OR pneumothoraces OR pneumothorax (MeSH) AND recurrence OR reoccurrence OR recurrent OR repeat OR predictor OR predictive]. Authors were not contacted directly. Reference lists of publications were then scrutinized to identify additional relevant studies. Duplicates were removed. Publications examining CT findings after an initial pneumothorax and recording pneumothorax recurrence as it related to these CT findings were considered. Case reports, qualitative reviews, articles lacking patient data, studies lacking information on recurrence and studies using investigations other than CT were excluded. We included studies that reported on the prevalence of blebs and bullae in patients with either PSP or SSP with follow-up data for recurrent pneumothorax. A total of 18 such PSP and 3 SSP studies were identified. From these, we extracted: patient demographics; therapeutic interventions for the pneumothorax; number and laterality of pneumothoraces; number and laterality of recurrences; numbers of patients with CT scan evidence of emphysematous changes with reference to the side of the initial pneumothorax and any recurrence; and follow-up durations. Where articles gave figures separately for ipsilateral and contralateral pneumothorax recurrence and the presence of bullae, these were recorded separately.

Where odds ratio (OR) for recurrence was not reported, we calculated the OR and 95% confidence intervals (95% CIs) based on the 2×2 contingency table for recurrence against presence of emphysematous lesions. Following the Haldane–Ascombe correction, zero values in cells were replaced with 0.5 to prevent zero or infinite values of the OR.^18^ Primary outcome was degree of association between pneumothorax recurrence and presence of CT abnormality. Secondary outcomes included association between recurrence laterality and CT abnormalities.

### Data analysis

Data were analysed and plotted using the Tidyverse^19^ and Meta^20^ packages software R^21^ and R studio.^22^ There was evidence for heterogeneity of effects between studies and so a random effects model was used to estimate the common OR and standard error. 

## Results

**Figure 1.**
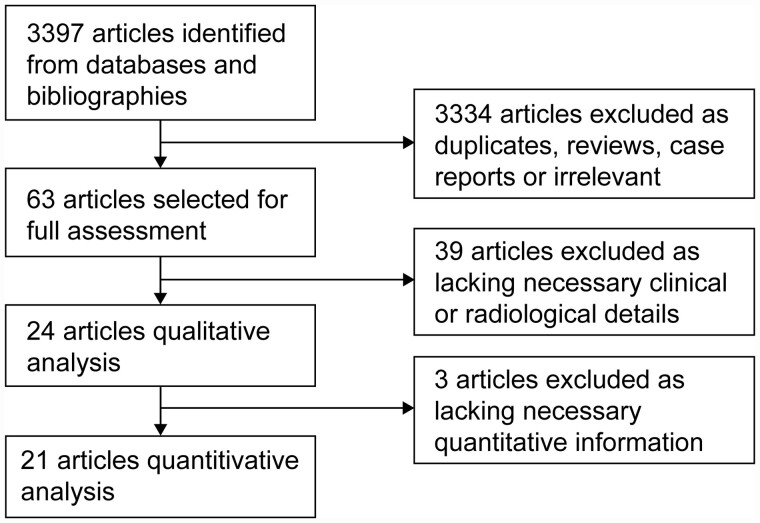
Study selection PRISMA diagram.

The initial search yielded 3397 articles. Two additional articles meeting the inclusion criteria were identified from the reference lists of screened articles. After exclusion of duplicates, 2586 articles were screened and 2523 were excluded as irrelevant or case reports or qualitative reviews (Figure 1). Of the remaining 63 articles, 39 lacked appropriate information on patient characteristics, recurrences or CT scans. In total, 24 studies were identified for quantitative analysis: 5 prospective^13–15^^,^^23^^,^^24^ and 19 retrospective reviews of notes^11^^,^^12^^,^^16^^,^^25–39^ (Table 1).

**Table 1.** Studies relating CT findings to pneumothorax recurrence: descriptive

	Mean age (range)/years	Type (*n*)	Study type	Imaging feature	Intervention (*n*)	Reference
Asai *et al.* (2008)	68.3 (45–85)	SSP (38)	Retrospective	CT bullae at AER	Surgery (38)	25
Asano *et al.* (2019)	<50	PSP (192)	Retrospective	CT blebs & bullae	Surgery (192)	38
Casali *et al.* (2013)	27 (14–41)	PSP (176)	Retrospective	CT severity score	Conservative (12) Intercostal drain (164)	27
Chen *et al.* (2016)	NR	PSP (553)	Retrospective	CT blebs & bullae	Surgery (425)	32
Hirai *et al.* (2007)	40.6 (15–98)	PSP (168)	Retrospective	CT blebs & bullae	Conservative (3) Intercostal drain (94) Surgery (62)	33
Huang *et al.* (2007a)	21.9 (NR)	PSP (213)	Retrospective	CT blebs & bullae	Surgery (178)	11
Huang *et al.* (2007b)	22.07 (NR)	PSP (102)	Retrospective	CT blebs & bullae	Conservative (1) Intercostal drain (101) Surgery (100)	36
Isaka *et al.* (2013)	NR (51–93)	SSP (86)	Retrospective	CT Goddard score	Pleurodesis (16) Surgery (86)	28
Martinez-Ramoz *et al.* (2007)	NR	PSP (55)	Prospective	CT blebs & bullae	NR	14
Mitlehner *et al.* (1982)	26.7 (NR)	PSP (35)	Prospective	CT blebs & bullae	Conservative (1) Intercostal drain (34) Surgery (3)	15
Nathan *et al.* (2010)	14.2 (NR)	PSP (25)	Retrospective	CT blebs	Conservative (10) Aspiration (8) Intercostal drain (12) Surgery (13)	29
Ng *et al.* (2020)	<18	PSP (23)	Retrospective	CT blebs & bullae	NR	39
Nishimoto *et al.* (2018)	75 (70–80)	SSP (17)	Retrospective	CT reticulation	Intercostal drain (7)	26
Noh *et al.* (2015)	23.85	PSP (285)	Retrospective	CT blebs & bullae	Conservative (7) Intercostal drain (70) Surgery (183)	34
Onuki *et al.* (2019)	17 (NR)	PSP (36)	Prospective	CT blebs & bullae	Surgery (36)	24
Ouanes-Besbes *et al.* (2007)	26.6 (NR)	PSP (80)	Prospective	CT severity score	Intercostal drain (80) Surgery (1)	13
Primavesi *et al.* (2015)	NR (16–50)	PSP (56)	Retrospective	CT severity score	Conservative (23) Surgery (33)	37
Park *et al.* (2019)	20.5 (NR)	PSP (299)	Retrospective	CT severity score	Conservative (65) Intercostal drain (234)	40
Seguier-Lipszyc *et al.* (2011)	<18	PSP (46)	Retrospective	CT blebs & bullae	Conservative (9) Intercostal drain (12) Surgery (8)	30
Sevink *et al.* (2017)	58.2	SSP (50)	Retrospective	CT Goddard score	Intercostal drain (50)	35
Sihoe *et al.* (2000)	29 (18–47)	PSP (28)	Retrospective	CT bullae	Surgery (28)	16
Smit H.J. *et al.* (2000)	<50	PSP (101)	Retrospective	CT blebs & bullae	NR	12
Warner *et al.* (1991)	36.34	PSP (26)	Prospective	CT blebs & bullae	Intercostal drain (26)	23
Young Choi *et al.* (2014)	16.82 (NR)	PSP (114)	Retrospective	CT severity score	Conservative (42) Intercostal drain (72)	31

SSP, secondary spontaneous pneumothorax; CT, computerized tomography scan; AER, azygoesophageal recess; PSP, primary spontaneous pneumothorax; NR, not recorded.

A total of 21 studies reported on recurrence, of which 17 reported on recurrence in patients with PSP and 4 reported on recurrence in patients with SSP. The total sample size was 2475 (2334 PSP and 141 SSP) (Table 2). Follow-up durations varied between 1.7 and 188.3 months.^40^ As anticipated, patients presenting with PSP were younger than those with SSP: mean ages 24 years (range 14–98) vs. 70 years (range 45–85). Most patients were male, total male:female ratio of 7.7:1 (1516:197); for PSP the ratio was 7.5:1 (1462:196), while all but one patient with SSP was male. Of those individuals with SSP, 88% had emphysema and 12% had pulmonary fibrosis.^25^^,^^26^^,^^28^ For those individuals for whom data were available, 7.5% of PSPs were treated initially conservatively, 33.5% were treated with an intercostal chest drain and 50.4% underwent primary surgery.^11–13^^,^^15^^,^^16^^,^^24^^,^^27^^,^^29–34^^,^^36–38^^,^^40^ For SSPs, no patients were treated conservatively, 5% had drainage alone while 88% underwent early surgery.^25^^,^^26^^,^^28^

**Table 2.** Characteristics of pooled studies

	PSP			SSP			Total		
	*n*	*N*	*n*/*N* (%)	*n*	*N*	*n*/*N* (%)	*n*	*N*	*n*/*N* (%)
Total patients	2334			141			2475		
Recurrence						^a^			
Either laterality (total per patient)	748	2334	32.0	22	141	15.6	770	2475	31.1
Either laterality when CT changes present (per lung)	561	2068	27.1	19	65	29.2	580	2133	27.2
Either laterality when CT changes absent (per lung)	133	1085	12.3	3	76	3.9	136	1161	11.7
Ipsilateral (total per patient)	573	2075	27.6	22	141	15.6	595	2216	26.9
Ipsilateral when CT changes present (per lung)	412	1363	30.2	19	65	29.2	431	1428	30.2
Ipsilateral when CT changes absent (per lung)	107	460	23.3	3	76	3.9	110	536	20.5
Contralateral (total per patient)	175	1340	13.1				175	1340	13.1
Contralateral when CT changes present (per lung)	149	715	20.8				149	715	20.8
Contralateral when CT changes absent (per lung)	26	635	4.1				26	635	4.1
CT changes (per lung)						^a^			
Ipsilateral	652	763	85.5	15	38	39.5	667	801	83.3
Contralateral	507	994	51.0				507	994	51.0
Unspecified	1205	1788	67.4	50	103	48.5	1255	1891	66.4
Any laterality	2364	3545	66.7	65	141	46.1	2429	3686	65.9

PSP, primary spontaneous pneumothorax; SSP, secondary spontaneous pneumothorax.

a Rates calculated from SSP articles are always ‘per patient’.

Total number from 21 studies, 18 PSP, 3 SSP.^11–16^^,^^24–34^^,^^37^^,^^39^^,^^40^

Age data only extractable from 13 studies, 11 PSP, 2 SSP.^11^^,^^13^^,^^15^^,^^16^^,^^24–27^^,^^29^^,^^31^^,^^33^^,^^34^^,^^40^.

Gender data only extractable from 16 studies, 14 PSP, 2 SSP.^11^^,^^13–16^^,^^24–27^^,^^29^^,^^31^^,^^33^^,^^34^^,^^37^^,^^40^

Treatment modality data only extractable from 18 studies, 15 PSP (2306 cases), 3 SSP (141 cases).^11^^,^^13^^,^^15^^,^^16^^,^^24–34^^,^^37^^,^^40^

‘Positive CT findings’ typically equated to the presence of blebs or bullae. Four studies formulated a unique ‘dystrophic severity scores’ by assigning scores to the type/size, number and distribution of blebs/bullae.^13^^,^^27^^,^^37^^,^^40^ Two other studies used the established Goddard scale.^28^^,^^35^^,^^41^ One study generated a score for the degree of emphysematous change in patients with idiopathic pulmonary fibrosis.^26^ Although, by definition SSP requires lung pathology, three SSP studies separated cases between those with or without specific CT findings.^25^^,^^26^^,^^28^ For example, positive CT findings were defined as having ‘bullae at the azygoesophageal recess’^25^ or using the Goddard classification score ≥7, see Discussion.^28^

**Figure 2.**
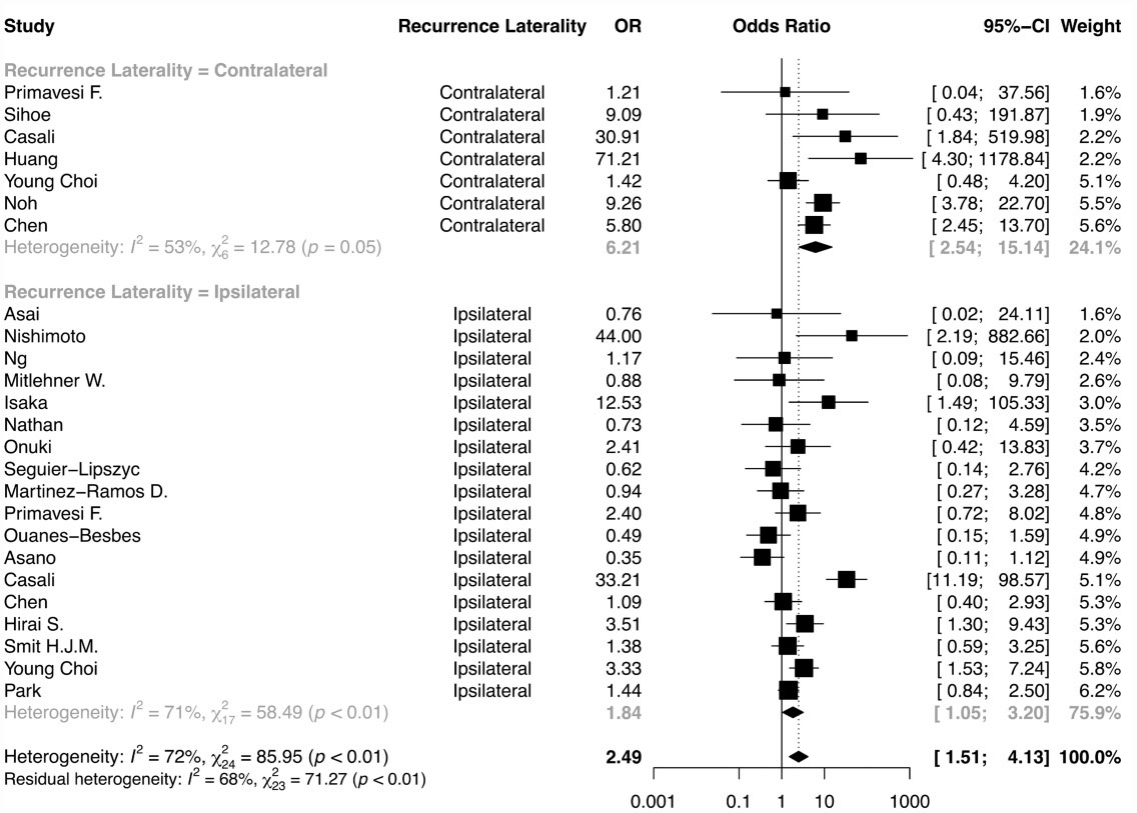
Forest plot comparing: CT changes present vs. absent, outcome: pneumothorax recurrence. OR, odds ratio; CI, confidence interval; recurrence laterality is relative to initial pneumothorax.

In pooled data from 2475 individuals, recurrence of pneumothorax was observed more often in those individuals reported to have ‘positive CT findings’ (OR 2.49, 95% CI 1.51–4.13; *P*<0.01) (Figure 2). We considered excluding those patients from three studies with SSP, but doing so had little effect on the positive association between CT changes and recurrence (OR 2.27, 95% CI 1.36–3.8; *P*<0.01; Supplementary Figure S1). A number of studies included children in their analysis potentially increasing heterogeneity.^15^^,^^27^^,^^29–31^^,^^33^^,^^34^ After exclusion of these seven studies, a positive relationship between CT changes and recurrence remained increasing our confidence in the association (OR 1.90, 95% CI 1.08–3.33; Supplementary Figure S2A).

It is plausible that patients undergoing surgery will differ from those not having surgery, either in terms of ongoing air leak, history of pneumothorax recurrence or frailty. Several studies included a mixture of patients treated surgically and without surgically. When we analysed studies in which allowed us to separate the two therapeutic approaches, the association between CT changes and recurrence appeared to persist for the non-surgery group (OR 1.65, 95% CI 1.13–2.41), but was no longer apparent for the publications involving surgery, which showed more heterogeneity (OR 2.07, 95% CI 0.43–9.95; Supplementary Figure S2B).^12^^,^^14^^,^^16^^,^^24^^,^^25^^,^^28^^,^^31^^,^^38^

In individual lungs with CT abnormalities the recurrence rate was 27%, but only 12% in lungs that were normal by CT scan (Table 2). It should be noted that per-lung recurrence rates will be lower than per-patient recurrence rates because their denominators differ. Overall, 31% of patients suffered a recurrent pneumothorax. While this is lower than reported in some previous reports,^5–7^ it might reflect the relatively high level of primary surgery (surgery after a first pneumothorax) in several published CT studies. When we focussed only on studies in which primary surgery was never performed, the overall recurrence was 41%.^12^^,^^14^^,^^31^^,^^40^ By contrast, the recurrence rate fell to 10% when studies in which all patients underwent primary surgery for pneumothorax.^16^^,^^24^^,^^25^^,^^28^^,^^38^

Overall, 66% of lungs from patients had CT evidence of emphysematous change, including blebs and bullae; 83% of these changes were ipsilateral to the initial pneumothorax and 51% were contralateral (Table 2). Ipsilateral recurrence of pneumothorax (27%) was twice as likely as contralateral (13%), and in both instances CT abnormalities were seen more often in cases that recurred: 30% suffered ipsilateral recurrence if CT changes were present vs. 21% without CT changes; 21% suffered contralateral recurrence if CT changes were present vs. 4% without CT changes.

In the absence of positive CT findings, recurrence was 3 times more likely for PSP cases than SSP cases (12% vs. 4%) (Table 2). However, unlike PSP, SSP is associated with a high rate of subsequent mortality,^42^ and so the lower rate of recurrence in the SSP group may reflect loss of patients owing to death. Nevertheless, when CT findings were present, the rate of recurrence was similar between PSP and SSP (27% and 29%, respectively). The association held true for PSP when considering emphysematous changes including bullae and blebs. Bullae at the azygoesophageal recess or increased Goddard score were associated with recurrent SSP, as shown by subgroup analysis. The incidence of reported positive CT findings was higher in PSP than in SSP at 67% vs. 46%, but the differing definitions of ‘positive CT finding’ in SSP and PSP make direct comparison difficult and so should be treated with caution.

We noted substantial between-study heterogeneity (*I*^2^=72%) and so we grouped studies according to the laterality of the bullae and laterality of the recurrence relative to the original pneumothorax. There was good evidence that the effect size differed between groups (Supplementary Figure S3). The effect size for ipsilateral recurrence was significantly different to contralateral recurrence (*P*<0.05). Contralateral recurrence was associated with CT change (OR 6.21, 95% CI 2.54–15.14), although ipsilateral recurrence was still positively associated with CT changes (OR 1.84, 95% CI 1.05–3.20) (Figure 2). Four studies reported the association of contralateral bullae with contralateral recurrence showing evidence of association (OR 8.11, 95% CI 4.47–14.69) with very low heterogeneity (Supplementary Figure S3). Ipsilateral recurrence was reported in association with ipsilateral bullae, bullae in either lung, or without information of bullae laterality. A ‘leave-one-out analysis’ identified two articles that contribute substantial heterogeneity. Within articles that reported contralateral recurrence (Figure 2), omitting Young Choi *et al.* reduced heterogeneity (*I*^2^) from 51% to <1% and increased the magnitude of association for contralateral recurrence from an OR of 6.21 (95% CI 2.54–15.14) to 8.12 (95% CI 4.58–14.42) (Figure 2). This may be because Young Choi *et al.* is the only article reporting contralateral recurrence that restricted its cohort to children. For articles reporting ipsilateral recurrence, omitting Casali *et al.* reduced heterogeneity (*I*^2^) from 71% to 45%, decreasing the magnitude of association for ipsilateral recurrence from an OR of 1.84 (95% CI 1.05–3.20) to 1.50 (95% CI 1.13–1.97). We were not able to identify study characteristics to explain this observation (Figure 2).

Some authors reported that specific features of the CT abnormalities were associated with recurrence.^13^^,^^14^^,^^23^^,^^25^^,^^31^^,^^40^ Bleb size,^23^^,^^31^^,^^40^ number^23^^,^^31^ and distribution were positively associated with recurrence in some reports,^13^^,^^14^^,^^25^^,^^31^^,^^40^ but these associations were not replicated in other studies of bleb size,^12–15^^,^^40^ number^13–15^^,^^40^ and distribution.^12^^,^^15^ Too few articles provided data in a format that would have permitted their inclusion in our analysis.

## Discussion

Our analysis shows that CT abnormalities predict both per-patient and per-lung recurrence of pneumothorax. Individuals with abnormalities identified on their thoracic CT scans were 2.5 times more likely to suffer a recurrence compare with those without (OR 2.49, 95% CI 1.51–4.13). Recurrence contralateral to the first pneumothorax was particularly strongly associated with contralateral CT change (OR 8.11 95% CI 4.47–14.69).

Overall, 66% of the lungs of patients who had suffered a pneumothorax had CT evidence of emphysematous change. When such CT changes were present, they were more likely to be ipsilateral to the presenting pneumothorax: 83% ipsilateral vs. 51% contralateral. Ipsilateral recurrence was 2-fold more likely than contralateral and was associated with the side of emphysematous change on CT. This supports a mechanistic link between emphysematous change and pneumothorax.

The observed recurrence rate of 10% following primary surgery is surprisingly high.^16^^,^^24^^,^^25^^,^^28^^,^^38^ In recent work, recurrence following video assisted thoracoscopic surgery (VATS) has been reported as low as 5%.^10^ In our meta-analysis, only five studies reported surgery in isolation with associated recurrence data and CT findings.^16^^,^^24^^,^^25^^,^^28^^,^^38^ These studies account for 380 individuals, with 37 recurrences reported (9.7%). The reason for this unexpectedly high recurrence rate is unclear but might reflect the reporting of contralateral pneumothoraces, which should be unaffected by unilateral surgery,^16^ or the exclusion of patients lost to follow-up, which was performed in one study in effort to avoid attrition bias.^24^

The low level of conservative management of pneumothoraces was striking in the published qualifying for this meta-analysis. In the UK, many patients with PSP are treated conservatively according to BTS guidelines.^8^ In one recent series, 30% (50/168) of PSPs were treated conservatively,^43^ contrasting with only 8% of the 2306 PSP cases reporting intervention in this meta-analysis. This may reflect national differences, as since UK and USA guidelines differ in their thresholds for intervention, with American guidance more interventionalist compared with the UK and other European countries.^8^^,^^44^ Biases may also have arisen from the relatively large number of series from surgical centres. Indeed, surgical intervention formed an inclusion criteria for four studies,^16^^,^^24^^,^^25^^,^^38^ although was an exclusion criterion for only two.^14^^,^^40^ The low level of conservative management of SSP was less surprising since failure to intervene in cases of SSP can have fatal consequences. That VATS was employed in 74% of interventions across SSP studies that reported surgical intervention^25^^,^^28^ is, however, at odds with typical UK experience where talc or blood patch pleurodesis is commonly used, and suggests that national differences and a degree of publication bias may skew the SSP literature.

Comparisons of radiological changes between studies are challenging. Publications varied in the detail supplied, especially whether pneumothoraces were ipsilateral to CT abnormalities.^12^^,^^14^^,^^29^^,^^30^^,^^33^ The definitions used for ‘positive CT findings’ also differed, e.g. the cut-off size of blebs vs. bullae being variously 1^11^^,^^13^^,^^16^^,^^27^^,^^31^^,^^37^^,^^40^ or 2 cm^15^^,^^34^ or not reported.^14^^,^^25^^,^^29^^,^^30^^,^^32^^,^^33^^,^^38^^,^^39^ A proportion of studies employed unique definitions of ‘positive CT findings’, occasionally developing bespoke severity scores,^13^^,^^26^^,^^27^^,^^40^ while some used the more established ‘Goddard scale’.^28^^,^^35^

In conclusion, we observe by meta-analysis a significant association between the recurrence of pneumothorax and features of emphysematous change on thoracic CT imaging. This association is particularly striking for contralateral pneumothoraces, although these are less common than ipsilateral recurrences overall. This meta-analysis has relevance for guidelines of pneumothorax management. Insufficient data are available to link specific CT features, such as lesion size or number, with recurrence and require further study.

## Supplementary material

Supplementary material is available at *QJMED* online.

## Funding 

S.J.M. was supported by the MRC (MR/R009120/1, G1002610), EPSRC (EP/R03558X/1), British Lung Foundation, Cambridge NIHR BRC and the Alpha1 Foundation (pC ID 614939 & pC ID 395467).

*Conflict of interest*. None declared. 

## Supplementary Material

hcab020_Supplementary_Data
