# Supplementary figures and images for: Meta-analysis of the association between emphysematous change on thoracic computerized tomography scan and recurrent pneumothorax
Source: QJM. 2021 Feb 4;115(4):215–21. doi: 10.1093/qjmed/hcab020 (PMC9020478; doi:10.1093/qjmed/hcab020)

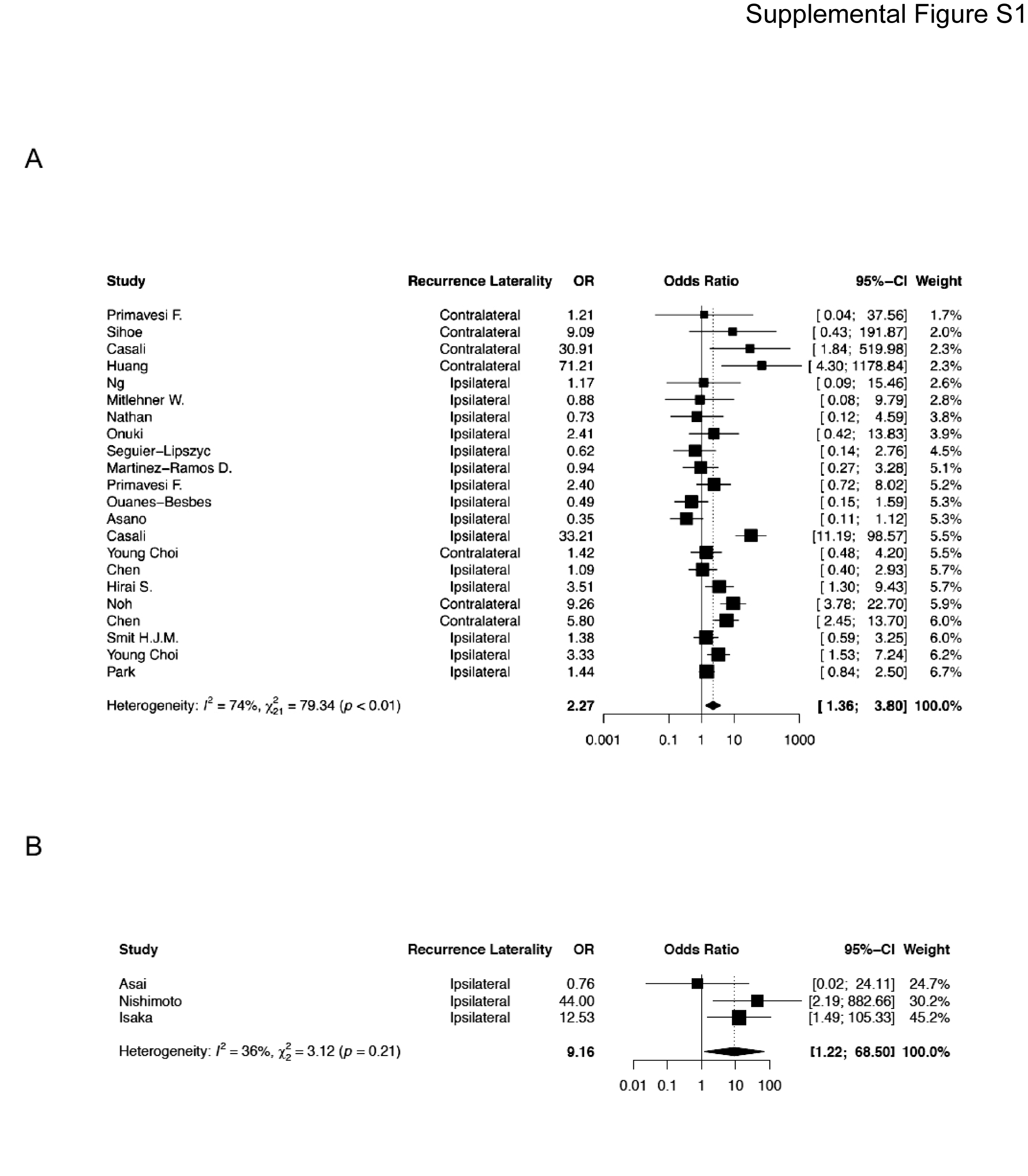

Supplement: hcab020_Supplementary_Data [file hcab020_supplementary_data.zip › FigS1.2.tif]

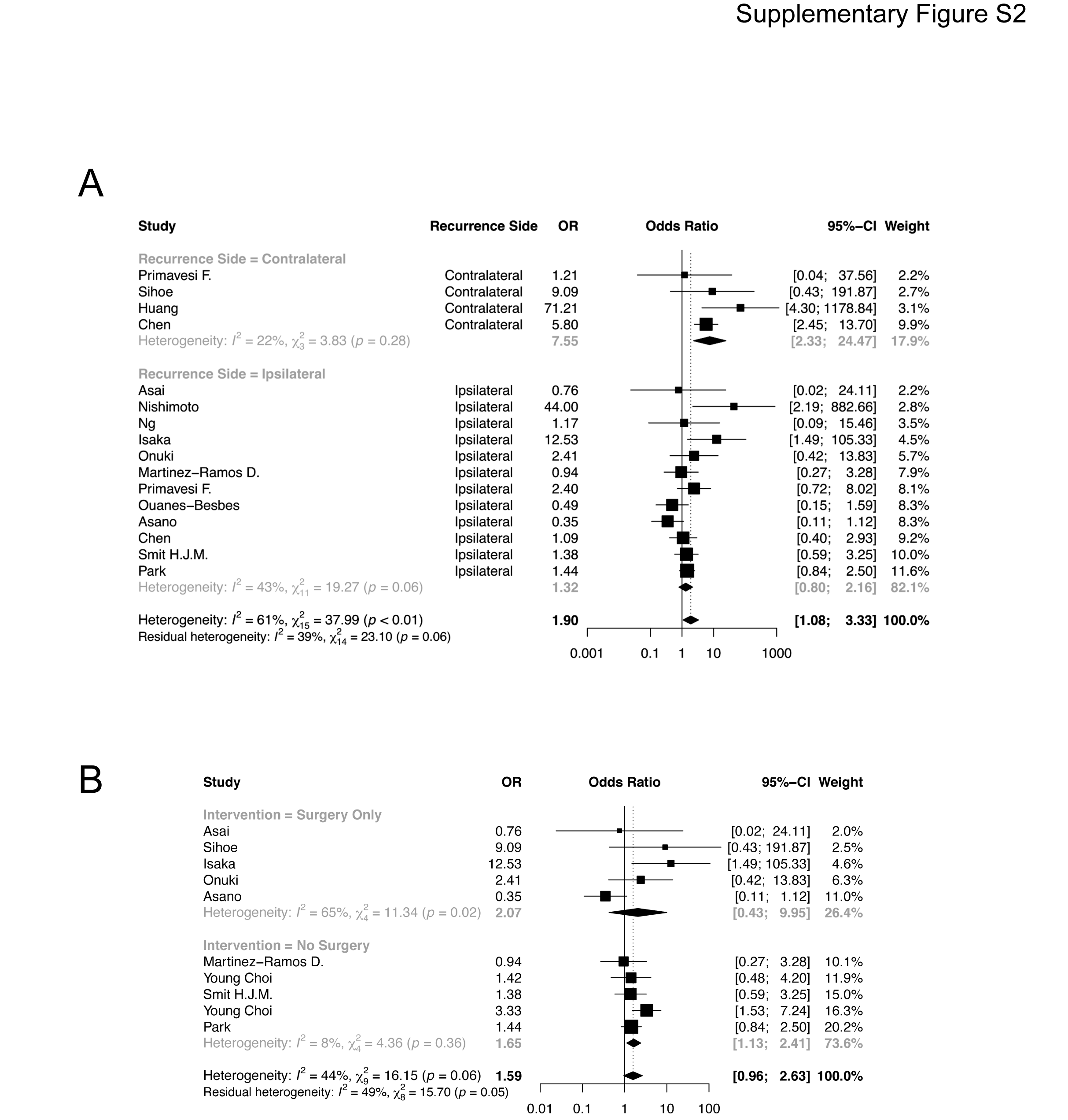

Supplement: hcab020_Supplementary_Data [file hcab020_supplementary_data.zip › FigS2.4.tif]

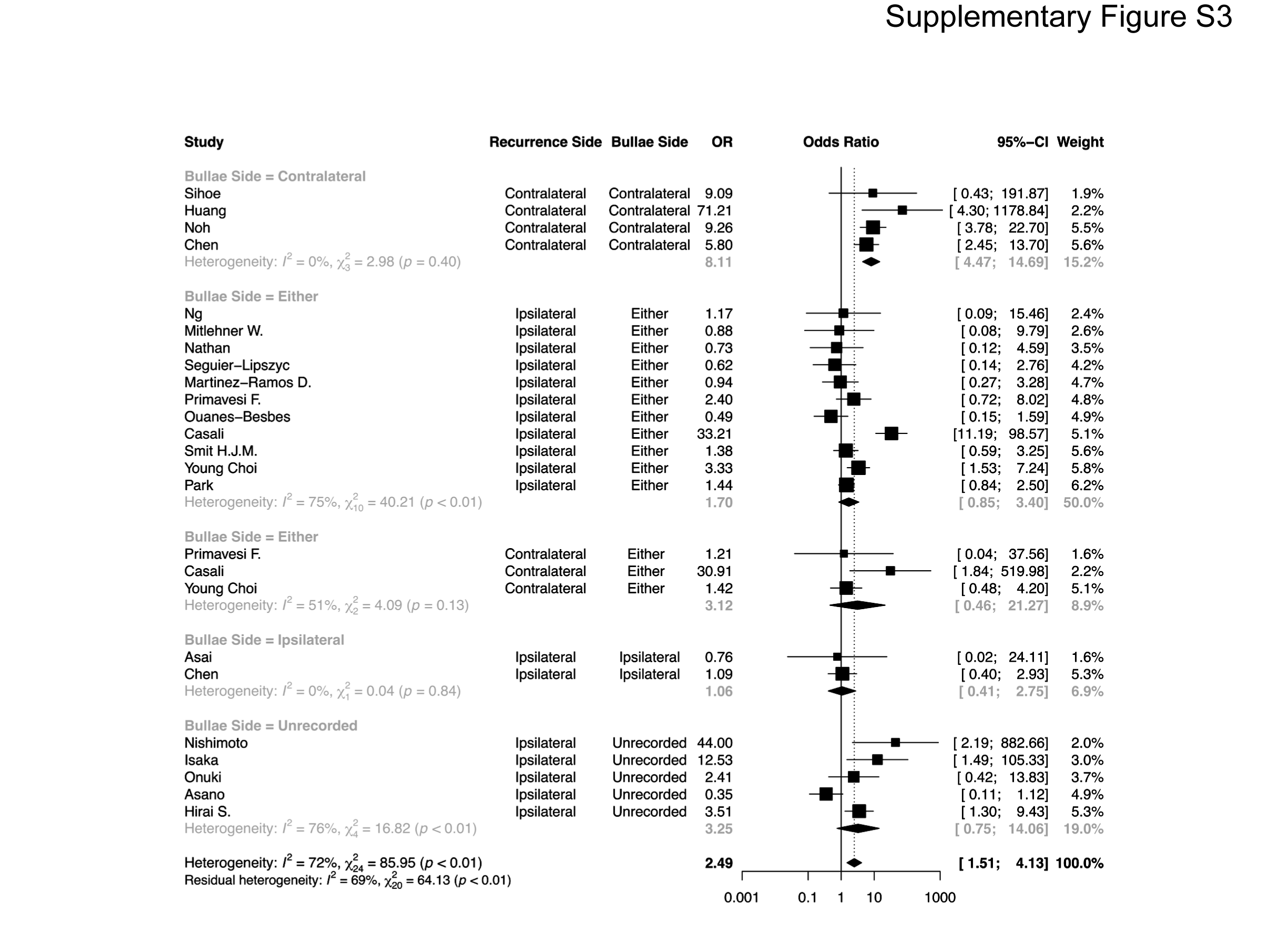

Supplement: hcab020_Supplementary_Data [file hcab020_supplementary_data.zip › FigS3.1.tif]
